# Exosomal mediators in sepsis and inflammatory organ injury: unraveling the role of exosomes in intercellular crosstalk and organ dysfunction

**DOI:** 10.1186/s40779-024-00527-6

**Published:** 2024-04-22

**Authors:** Ting Gong, You-Tan Liu, Jie Fan

**Affiliations:** 1 Department of Surgery, University of Pittsburgh School of Medicine, Pittsburgh, PA 15213 USA; 2 Department of Anesthesiology, Shenzhen Hospital, Southern Medical University, Shenzhen, Guangzhou, 518110 China; 3 Research and Development, Veterans Affairs Pittsburgh Healthcare System, Pittsburgh, PA 15240 USA; 4 Department of Immunology, University of Pittsburgh School of Medicine, Pittsburgh, PA 15213 USA; 5 McGowan Institute for Regenerative Medicine, University of Pittsburgh, Pittsburgh, PA 15219 USA

**Keywords:** Sepsis, Exosomes, Intercellular crosstalk, Inflammation, Biomarkers

## Abstract

Sepsis, a severe systemic inflammatory response to infection, remains a leading cause of morbidity and mortality worldwide. Exosomes, as mediators of intercellular communication, play a pivotal role in the pathogenesis of sepsis through modulating immune responses, metabolic reprogramming, coagulopathy, and organ dysfunction. This review highlights the emerging significance of exosomes in these processes. Initially, it provides an in-depth insight into exosome biogenesis and characterization, laying the groundwork for understanding their diverse and intricate functions. Subsequently, it explores the regulatory roles of exosomes in various immune cells such as neutrophils, macrophages, dendritic cells, T cells, and B cells. This analysis elucidates how exosomes are pivotal in modulating immune responses, thus contributing to the complexity of sepsis pathophysiology. Additionally, this review delves into the role of exosomes in the regulation of metabolism and subsequent organ dysfunction in sepsis. It also establishes a connection between exosomes and the coagulation cascade, which affects endothelial integrity and promotes thrombogenesis in sepsis. Moreover, the review discusses the dual role of exosomes in the progression and resolution of sepsis, exploring their complex involvement in inflammation and healing processes. Furthermore, it underscores their potential as biomarkers and therapeutic targets. Understanding these mechanisms presents new opportunities for novel interventions to mitigate the severe outcomes of sepsis, emphasizing the therapeutic promise of exosome research in critical care settings.

## Introduction

Sepsis, a life-threatening condition, is characterized by organ dysfunction stemming from a dysregulated host response to infection [1], which remains a predominant cause of mortality in intensive care units (ICUs) globally [2]. The progression of sepsis initially involves a transition from mild to severe stages, eventually culminating in septic shock. This sequential advancement triggers abnormal immune system responses, disrupts cellular and tissue metabolism, causes coagulopathy, alters circulation, and ultimately results in multi-organ damage [3]. While sepsis is treatable and timely targeted interventions can improve prognosis [4], the intricate mechanisms underlying its onset, progression, and varied patient outcomes are not fully understood.

Exosomes are small extracellular vesicles that play a vital role in the cellular landscape, particularly in the context of sepsis. Ranging from 30 to 150 nm in size, these vesicles serve as natural carriers for various signaling molecules such as proteins, DNA, and microRNAs (miRNAs), which are small non-coding RNAs (ncRNAs) that hold a crucial position in regulating gene expression [5, 6]. Their unique ability to traverse bodily fluids without succumbing to enzymatic degradation makes them essential for the intercellular exchange of material and signaling. The cargo of exosomes, including proteins, lipids, and nucleic acids, is selectively packaged to deliver specific messages to target cells. Exosomes are crucial in intercellular communication as they carry specific molecules that reflect the state and nature of the parent cells. In sepsis, exosomes are pivotal in processes related to tissue and organ injury, inflammatory responses, and immune reactions [7]. They modulate the immune microenvironment by promoting cellular oxidative stress, immune cell aggregation, and the release of inflammatory cytokines. These actions ultimately affect the fate of cells and tissues [8, 9].

Under pathological conditions such as sepsis, the composition of exosomes varies significantly from that in normal physiological conditions. Cells of origin can precisely modulate the content of exosomes, selectively loading them with specific active substances. These loaded exosomes then transport these active substances to recipient cells, altering their phenotype and functions [9]. The clinical importance of exosomes in sepsis, specifically regarding their roles as biomarkers and mediators of ncRNAs, has been extensively reviewed in prior studies [10, 11]. Additionally, there has been research focusing on how the contents of exosomes influence cellular signaling pathways in sepsis and contribute to organ injury [12]. However, our review primarily concentrates on providing a more in-depth mechanistic insight from a microscopic to a holistic perspective. We delve into the role of exosome-mediated intercellular crosstalk in causing organ damage, and the potential value of addressing these scientific questions to apply exosomes in the clinical diagnosis and treatment of critically ill patients with infections.

This review offers a thorough examination of exosome biogenesis and its characteristics, laying the groundwork for understanding their diverse and intricate functions. We will scrutinize the regulatory effects of exosomes on various immune cells, a process that aids in elucidating their critical role in modulating immune responses, thereby enhancing our understanding of sepsis pathophysiology. The cargo of exosomes is rich in bioactive molecules that can induce metabolic alterations, characterized by the dysregulation seen in sepsis. They are also implicated in the coagulation cascade, impacting endothelial integrity, and promoting thrombogenesis. In the context of organ dysfunction, the role of exosome-mediated intercellular communication is of paramount importance. Additionally, this review explores the dual role of exosomes in the progression and resolution of sepsis, examining their complex involvement in both inflammatory and healing processes. Moreover, the potential of exosomes as biomarkers and therapeutic targets is highlighted.

## Exosome biogenesis and characterization

Exosomes are produced through a complex process that initiates within the cell’s endosomal system. It begins with the endocytosis of the plasma membrane, leading to the formation of early sorting endosomes. These structures evolve into late-sorting endosomes, which subsequently invaginate to generate multivesicular bodies (MVBs) containing intraluminal vesicles (ILVs) [13]. Depending on the cellular context, MVBs can either fuse with lysosomes for degradation or with the plasma membrane, releasing ILVs as exosomes into the extracellular milieu [14, 15]. Several molecular components work together to coordinate this complex process. For instance, Rab proteins regulate vesicular traffic, while the endosomal sorting complex required for transport and its constituents are essential for MVB and ILV formation. Tetraspanins, a group of transmembrane proteins, induce membrane curvature, facilitating vesicle formation [16]. Moreover, certain lipids, notably ceramides produced by enzymes like sphingomyelinase, play a fundamental role in this process [17]. The unique characteristics of exosome formation and release render them pivotal in mediating inflammatory responses and intercellular communication in sepsis [10].

Many techniques have been applied to characterize exosomes [18]. For example, nanoparticle tracking analysis, dynamic light scattering, and tunable resistive pulse sensing primarily focus on determining the exosomal size [19]. On the other aspect, transmission electron microscopy, localized surface plasmon resonance, and atomic force microscopy biosensors provide detailed insights into the morphology of exosomes [20–22]. Flow cytometry is utilized to evaluate the surface proteins of exosomes, shedding light on their size and structure [23]. In addition, sequencing, microarray analysis, and digital droplet PCR allow for a comprehensive analysis of exosomal cargo, including RNA [24].

Exosomes have emerged as promising candidates for biomarkers and therapeutic targets across a range of diseases, including cancer, neurodegenerative disorders, and cardiovascular conditions [25–28]. In sepsis, exosomes have shown significant potential as biomarkers [10], a topic we will explore extensively in subsequent sections. The molecular cargo of exosomes provides insight into the onset, progression, and potential treatments of disease by reflecting the status of the originating cells. The inherent stability of exosomes in bodily fluids, such as blood and urine, underscores their potential as non-invasive diagnostic tools. Exosomes have diverse biogenesis and clinical implications, and hold the promise to revolutionize medical research as their mysteries are uncovered.

## Exosomal mechanisms in sepsis-related immune regulation

**Fig. 1:**
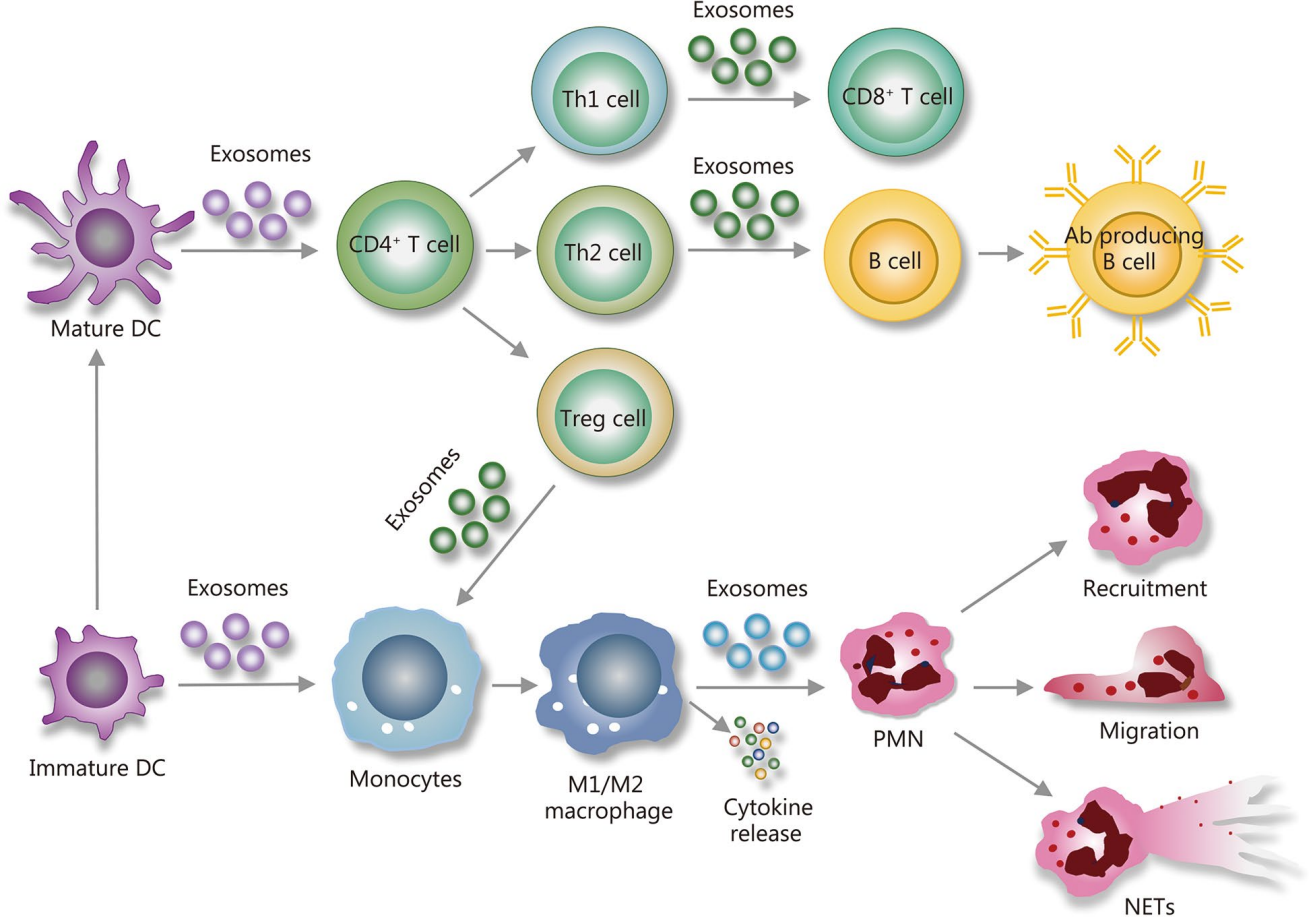
Mechanisms of exosome regulation of immunity in sepsis. Exosomes derived from immune cells are instrumental in orchestrating a balance between immunoregulatory and autoimmune responses within a complex network of immune interactions. These vesicles are rich in bioactive molecules and play key roles in the inflammatory process by guiding PMN recruitment and migration, supporting the innate immune response, and influencing macrophage polarization towards either pro-inflammatory M1 or anti-inflammatory M2 states, thereby shaping the progression of inflammation. DC-derived exosomes are crucial in engaging memory T cells, triggering their differentiation into Th1, Th2, or Treg cells and fostering a proliferative response essential for robust immunity. Additionally, these exosomes enhance adaptive immunity by aiding in B cell maturation and improving antigen presentation. Simultaneously, they significantly impact the activation and proliferation of CD8^+^ T cells, highlighting their extensive involvement in modulating immune responses, particularly during sepsis. PMN neutrophil, DC dendritic cell, NET neutrophil extracellular traps, Th1 type 1 helper T cells, Th2 type 2 helper T cells, Ab antibody

Exosomes, as key mediators in the immune system, play a pivotal role in the immune response during sepsis - from the initial activation of the innate immune system to the later stages of immune regulation. These vesicles are indispensable for transmitting immune signals and exert a profound influence on the behavior of immune cells such as neutrophils (PMNs), macrophages, dendritic cells (DCs), T cells, and B cells (Fig. 1; Table 1). Through their diverse molecular cargoes, exosomes contribute to the regulation of immune responses, including the modulation of cytokine production, antigen presentation, and immune cell activation.

**Table 1:** The association between exosomes and function of different immune cells

Donor cells	Exosomal cargo	Recipient cells	Function	Reference
Macrophages, DCs	Leukotriene biosynthetic enzymes	PMNs	Induction of PMN migration	[29]
Platelets	12-lipoxygenase, secretory phospholipase A2-IIA	PMNs	Pro-inflammatory responses	[30]
Macrophages	NADPH oxidase-derived ROS	PMNs	Triggering PMN necroptosis, amplification of inflammation	[31]
PMNs	miR-30d-5p	Macrophages	Induces macrophage pyroptosis in ALI	[32]
MSCs	miR-21-5p	Macrophages	Regulates inflammatory response, promotes repair after myocardial injury	[33, 34]
DCs	MHC-peptide complexes, co-stimulatory molecules	T cells, DCs	Antigen presentation, activation of memory, and naive T cells	[35–38]
APCs	Specific antigens	T and B cells	Stimulation of T and B cells, development of immune memory	[39, 40]
MSCs	miRNAs	T cells	Modulates proliferation and activation, phenotype shifts, cytokine reduction	[41, 42]
T cells	tRNA fragments, immunoregulatory cytokines	DCs, T cells	Regulates immune response, activation of antiviral pathways, feedback inhibition	[43, 44]

*DCs* dendritic cells, *PMNs* neutrophils, *MSCs* mesenchymal stem cells, *APCs* antigen-presenting cells, *tRNA* transfer RNA, *ROS* reactive oxygen species, *ALI* acute lung injury, *MHC* major histocompatibility complex, *NADPH* nicotinamide adenine dinucleotide phosphate

### Exosomes regulation of PMN recruitment and migration

Exosomes derived from immune cells contain a variety of bioactive substances, playing multiple roles during the inflammatory process. Those released from macrophages and DCs are known to carry leukotriene biosynthetic enzymes and act as potent inducers of PMN migration, underscoring their chemotactic influence on inflammation [29]. Intriguingly, the platelet-derived exosomes can be internalized by PMNs, and through the synergistic actions of 12-lipoxygenase and secretory phospholipase A2-IIA, they manifest pro-inflammatory responses [30]. Furthermore, in the context of hemorrhagic shock, activated macrophages release exosomes which induce the production of reactive oxygen species (ROS) within PMNs, primarily sourced from nicotinamide adenine dinucleotide phosphate (NADPH) oxidase. The surge of ROS triggers PMN necroptosis and amplifies the inflammatory response [31].

A recent animal study has revealed a potential therapeutic strategy for sepsis. By systemically delivering super-repressor IκB-loaded exosomes (Exo-srIκB), it was observed that these exosomes significantly reduced PMN infiltration and suppressed macrophage release of inflammatory cytokines such as tumor necrosis factor (TNF)-α, interleukin (IL)-1β, and IL-6 [45]. Thus, manipulating the bioactive molecules carried by exosomes represents a potential to alter the overall immune response in sepsis.

### Exosomes regulation of macrophage inflammatory responses

Macrophages are versatile immune cells and can exhibit both pro-inflammatory and anti-inflammatory characteristics depending on the molecular signals they receive. Recent studies have highlighted the critical role of exosomal miRNAs in regulating macrophage polarization and function. For example, mammary epithelial cell-derived exosomal miR-221 mediates M1 macrophage polarization via suppressor of cytokine signaling 1 (SOCS1)/signal transducer and activator of transcription proteins (STATs) to promote inflammatory response [46]. The exosomal miR-374b-5p from tubular epithelial cells (TECs) promotes M1 macrophage activation and worsens renal ischemia/reperfusion injury [47]. PMN-derived exosomal miR-30d-5p induces M1 macrophage polarization and triggers macrophage pyroptosis in sepsis-related acute lung injury (ALI) [32]. Furthermore, exosomes from the serum of septic mice transfer miR-155 to macrophages and therefore, promote M1 polarization by activating the nuclear factor-kappa B (NF-κB) pathway and suppressing SRC homology 2-containing inositol phosphatase 1 (SHIP1) and SOCS1, leading to increased macrophage proliferation [48]. Additionally, miR-155 carried by serum exosomes, worsens inflammation in the nervous system by triggering the growth and activation of microglia and astrocytes in lipopolysaccharide (LPS)-treated mice [49].

Mesenchymal stem cell-derived exosomes (MSC-Exos) regulate the M2 polarization of macrophages and inflammatory response via miR-21-5p to promote repair after myocardial injury [33]. MSC-Exo treatment modulated NF-κB and phosphoinositide 3-kinase/protein kinase B (PI3K/Akt) signaling pathways by inhibiting tumor TNF-associated factor 1 expression and enhancing macrophage M2 polarization [34]. These findings highlight the multifaceted roles of exosomal miRNAs in macrophage polarization and inflammatory responses, potentially leading to an increase in the M2-like phenotype.

In summary, exosomal miRNAs are significant regulatory molecules that modulate various signaling pathways in macrophages, a concept primarily demonstrated through animal model studies. Their impact on macrophage function, which can either amplify or attenuate the inflammatory response, underscores their potential therapeutic implications in inflammatory conditions such as sepsis. It is important to note that much of the current understanding in this area is derived from animal studies. To date, there are limited human trials exploring the role of exosomal miRNAs in macrophage regulation, emphasizing a critical gap between experimental models and clinical application.

### Role of DC-derived exosomes in the regulation of immunity

DCs play a vital role in the immune system as antigen-presenting cells (APCs) and bridge the gap between innate and adaptive immunity by capturing, processing, and presenting antigens to T cells. DCs secrete exosomes that carry major histocompatibility complex (MHC)-peptide complexes, which can directly bind to and activate memory CD4^+^ and CD8^+^ T cells. However, to initiate the activation of naive T lymphocytes, the exosomes need to be first captured by other DCs. Interestingly, these DCs are not required to express the corresponding MHC molecules themselves but can present the MHC-peptide complexes carried by the exosomes to specific T cells [35]. Additionally, DC-secreted exosomes are rich in MHC and T cell co-stimulatory molecules, which activate antigen-specific naive CD4^+^ T cells [36]. The composition of the exosomes, which includes MHC I and MHC II molecules, adhesion molecules, co-stimulatory molecules, heat-shock proteins, and tetraspanins, endows them with extensive immunomodulatory abilities [37–39].

Furthermore, exosomes from mature DCs have shown to be more effective in inducing T cell activation in vitro and eliciting effector T cell and antibody responses in vivo, compared to those from immature DCs [35, 50]. In addition to these functions, exosomes released by DCs not only induce the maturation and differentiation of immature DCs and monocytes [51], but also facilitate the differentiation of T cells into various subtypes including type 1 helper T cells (Th1), type 2 helper T cells (Th2), and regulatory T (Treg) cells [40, 52]. The diverse functions of DC-released exosomes underscore their central role in modulating immune responses and maintaining immune homeostasis.

### Exosomes regulation of T cell responses

Exosomes can transport specific antigens that stimulate T and B cells, thereby leading to their activation and the development of long-term immune memory [39, 40]. This creates a state of readiness for a rapid and specific response to the same pathogens in future encounters. Traditional antigen presentation requires APCs to process antigenic peptides into MHC-peptide complexes. These complexes, along with co-stimulatory molecules, bind to the T cell receptors, subsequently driving the activation and proliferation of T cells. Exosomes offer a more streamlined approach, as they are capable of presenting antigens directly to T cells without the conventional APC mediation or the need for reprocessing into MHC-peptide complexes [50]. This mode of action significantly enhances the efficiency of antigen presentation, which is beneficial for initiating swift immune defenses against pathogens.

In the absence of active APCs, it has been observed that naive CD8^+^ T cells can recognize exosomes that are shed from APCs. These exosomes, particularly those expressing intercellular adhesion molecule-1 and B7, are highly immunogenic. They can stimulate proliferative responses in CD8^+^ T cells and promote their differentiation into effector T cells, which are critical for immediate immune responses [39]. Additionally, MSC-Exos have been reported to modulate the proliferation and activation of various T cell subsets. They can influence the immune response by inducing a shift from Th1 to Th2 phenotype, reducing the differentiation potential of T cells towards Th17 cells, and decreasing the release of pro-inflammatory cytokines like interferon (IFN)-γ [41, 42].

The exosomes released from T cells also function as immunoregulatory entities in regulating DCs by activating the cyclic GMP-AMP synthase/stimulator of interferon genes (cGAS/STING) pathway and triggering the activation of IFN regulatory factor 3 (IRF3)-dependent genes that regulate IFN [43, 44]. This activation strengthens the antiviral responses of DCs, highlighting the intricate communication between innate and adaptive immune cells [43]. Furthermore, activated T cells are capable of secreting exosomes containing specific transfer RNA fragments. Under certain circumstances, these fragments can suppress further activation of T cells, providing a feedback mechanism that ensures the immune response remains within necessary levels [44]. The stability of immunoregulatory cytokines encapsulated within T cell-derived exosomes is notably higher compared to their free counterparts, suggesting exosomes as promising vehicles for targeted drug delivery and potential therapeutic tools.

### Exosomes regulation of B cell activation and antigen presentation

In the realm of B cell-mediated immunity, plasma exosomes have been identified as efficient antigen carriers. These exosomes exhibit a remarkable ability to bind with B cells, enhance antigen presentation, and avoid degradation that is typically associated with free proteins. This interaction can induce the production of autologous antibodies and trigger the activation of T cells, potentially serving as a decoy against complement-mediated cytotoxicity [53]. By directly presenting antigens to B cells, exosomes efficiently stimulate B cells to mature and differentiate into plasma cells that produce antibodies. These activated B cells, in turn, can act as APCs, presenting antigens to T cells, particularly CD4^+^ T helper cells [54, 55]. Upon antigen recognition by CD4^+^ T cells, a cascade of events leads to their activation and differentiation into Th1 cells, which are instrumental in directing the immune response towards a Th1-type profile. This differentiation process results in the production of cytokines such as IFN-γ, which is pivotal in enhancing the body’s ability to defend against intracellular pathogens [55].

Additionally, RNA exosomes have been identified as crucial within B cells for their involvement in RNA processing, gene expression regulation, genomic stability, and in shaping B cell differentiation and maturation processes. These functions of RNA exosomes are essential in the formation of immune memory, thereby having a fundamental impact on maintaining the normal function of the immune system [56–58]. Recent studies underscore the centrality of the RNA exosome in these processes [24, 59]. B cell receptor expression and subsequent antibody secretion rely on successful V(D)J [variable (V), diversity (D), and joining (J)] recombination. RNA exosomes contribute to this critical function by resolving ncRNAs and non-B DNA structures, while maintaining an open chromatin state that is conducive to recombination-activating gene (RAG) recombinase access and successful V(D)J recombination. Impairment in RNA exosome function can lead to the accumulation of ncRNAs and non-B DNA structures near the V(D)J locus, causing chromatin closure and hindering RAG recombinase activity. This disruption can potentially block the D to JH recombination, triggering a p53-mediated apoptotic pathway, resulting in the death of pro-B cells, and ultimately, compromising the development and functionality of B cells [24, 59]. This interconnected network of exosome-mediated processes exemplifies the complexity and sophistication of immune regulation.

## Exosome modulation of sepsis-induced metabolic alterations

During the acute phase of infection, there is a metabolic shift from oxidative phosphorylation (OXPHOS) to aerobic glycolysis (known as the Warburg effect) serves as a crucial mechanism of host defense [60]. Despite an ample supply of oxygen, cells in this phase favor energy production through glycolysis over the more efficient OXPHOS. This preference for glycolysis, termed “metabolic reprogramming” or aerobic glycolysis, can be advantageous during the initial stages of inflammation as it boosts the production of metabolic intermediates necessary for cellular biosynthesis and bioenergetics, facilitating cell growth, proliferation, and differentiation [61]. Over time, however, if mitochondrial function is compromised, the cells may struggle to effectively restore OXPHOS and metabolic balance, leading to a state of “cellular pathogenic hypoxia” where they are unable to optimally utilize oxygen [62].

Recent research has shown that immune cells in sepsis exhibit distinct metabolic characteristics that impact their immune function. The metabolic states of M1 and M2 differ from those of resting macrophages; such metabolic reprogramming is vital for macrophage activation and function [63]. M1 macrophages increase glucose uptake and lactate secretion while reducing oxygen consumption. In contrast, M2 macrophages are primarily reliant on the OXPHOS pathway [64].

Mitochondrial dysfunction plays a pivotal role in the metabolic reprogramming associated with sepsis [61]. Damage to mitochondria can shift cellular energy production towards anaerobic glycolysis to meet ATP demands [62]. The exchange of mitochondrial elements through exosomes can thus profoundly influence the metabolic and functional states of target cells [65]. A study has shown the therapeutic potential of exosomes sourced from adipose-derived mesenchymal stem cells (AdMSC-Exos). These vesicles have been shown to mitigate mitochondrial ROS stress in macrophages stimulated with LPS, enhancing mitochondrial integrity and the efficiency of OXPHOS, thereby orchestrating a metabolic shift in macrophages—from the pro-inflammatory M1 phenotype towards the anti-inflammatory M2 phenotype [66].

Inhibition of the mitochondrial respiratory chain is associated with an increase in ROS production. This has elucidated the significant correlation between OXPHOS inhibition and ROS generation [67]. Further research has shown that exosomes from adipose-derived stem cells reduce ROS accumulation in macrophages by modulating the expression of nuclear factor erythroid 2-related factor 2/heme oxygenase-1 (Nrf2/HO-1), thus promoting polarization towards an M2 phenotype [68]. AdMSC-Exos also facilitate macrophage polarization to the M2 phenotype through the activation of the sphingosine-1-phosphate/sphingosine kinase 1/sphingosine-1-phosphate receptor 1 (S1P/SK1/S1PR1) signaling pathway, thus ameliorating cardiac injury following myocardial infarction [69].

Exosomes affect cell metabolic reprogramming through their role in intercellular communication. Exosomes transport genomic and mitochondrial DNA, which function as signaling molecules involved in the cGAS/STING cytosolic DNA sensing pathway and modulate gene expression via inducing IFN-stimulated genes through an IRF3-dependent mechanism [43]. Consequently, this communication prompts a change in the metabolic programming of DCs, boosting their capacity to combat viral infections and underscoring the importance of intercellular signaling in immune responses and pathogen defense.

By upregulating the expression of IL-1 receptor-associated kinase-M, a negative regulator of the Toll-like receptor (TLR) signaling pathway, hypoxia-inducible factor-1α (HIF-1α) has been recognized as a pivotal regulator in modulating the transition of blood monocytes from a pro-inflammatory to an immunosuppressive state in septic patients [70]. Hypoxia induces the overexpression of HIF-1α in renal TECs, which then promotes the release of exosomal miR-23a, reprogramming macrophages to a pro-inflammatory phenotype by inhibiting the ubiquitin-editing enzyme A20 [71]. Serum-derived exosomes, which carry miR-155 and target the suppressors SHIP1 and SOCS1, facilitate glycolysis and inflammatory responses in septic mice by upregulating the STAT3/HIF-1α axis [48, 72]. Study indicates that aerobic glycolysis induced by the Akt-mammalian target of rapamycin (mTOR)-HIF-1α pathway is fundamental to the reprogramming of cellular metabolism [73]. During sepsis, platelet-derived exosomes carrying high mobility group box 1 (HMGB1) exacerbate vascular injury and myocardial dysfunction by promoting the formation of neutrophil extracellular traps (NETs) via the Akt/mTOR pathway [74].

In sepsis, there is a marked increase in lactate due at least in part to impaired clearance. Lactate serves as a classical biomarker of poor prognosis in sepsis, with its levels correlating significantly with disease severity, morbidity, and mortality [75]. Macrophages can take up extracellular lactate through monocarboxylate transporters, and lactate-driven p300/ cAMP response element-binding (CREB) protein-mediated acylation of HMGB1 promotes the release of HMGB1-containing exosomes from macrophages, leading to endothelial dysfunction [76]. These findings suggest that exosome-mediated metabolic reprogramming is a key feature of immune cell activation.

These findings provide novel insights into the metabolic alterations induced by sepsis and offer a fresh perspective for further research. The role of exosomes in metabolic regulation reveals the intricate mechanisms employed by immune cells in responding to infections. By modulating the metabolic states of immune cells, exosomes have the potential to become powerful tools in the future for treating sepsis.

## Exosomes regulation of sepsis-induced coagulopathy and endothelial dysfunction

**Fig. 2:**
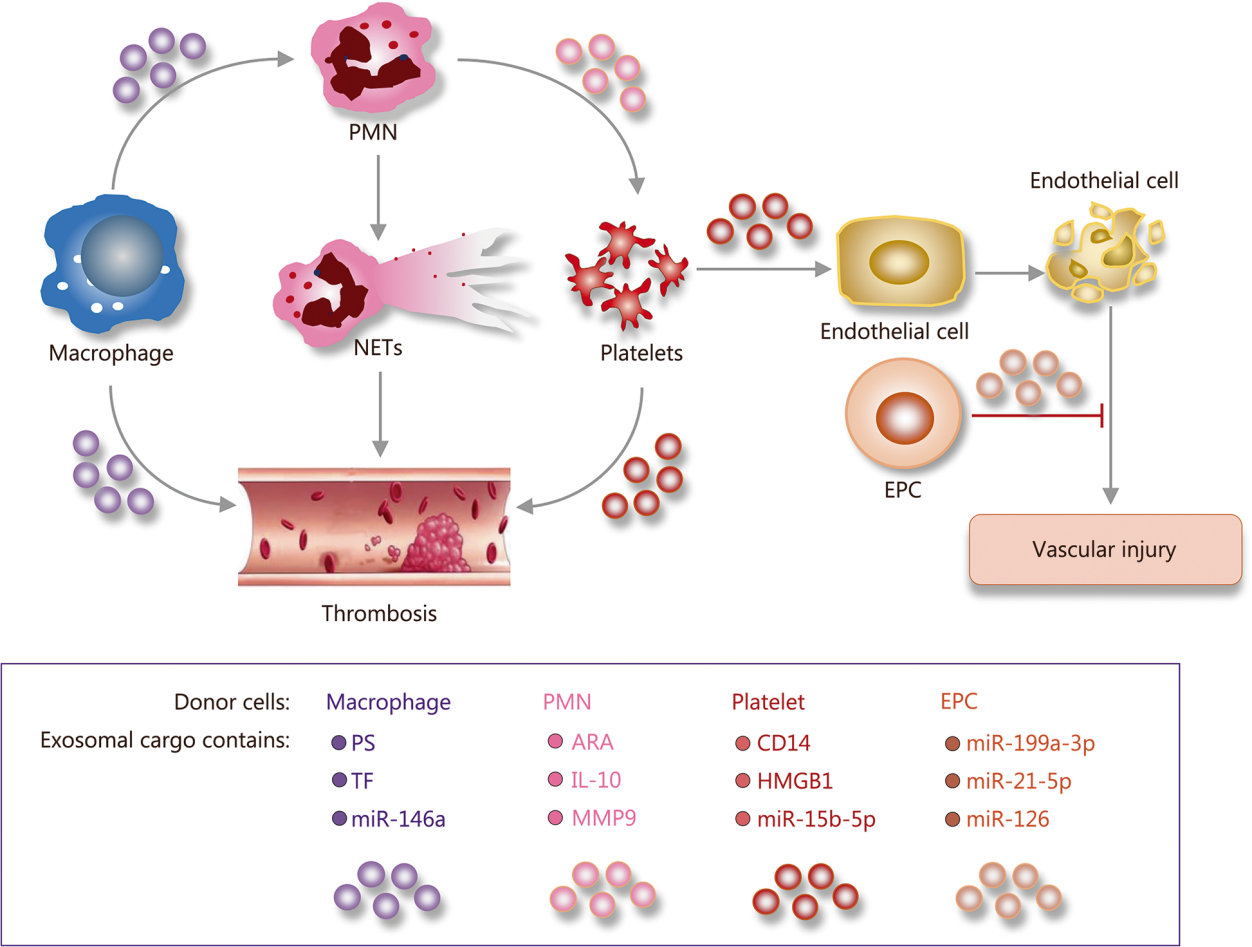
Exosomes regulation of sepsis-induced coagulopathy. Exosomes intricately regulate the balance between immune and coagulation responses in the pathophysiology of sepsis-induced coagulopathy. Macrophage-derived exosomes directly exert procoagulant activity, thereby initiating the coagulation cascade. Furthermore, these vesicles significantly influence neutrophil (PMN) recruitment and neutrophil extracellular traps (NETs), effectively linking inflammatory and coagulation pathways. Additionally, platelet-derived exosomes enhance both coagulation and inflammation, which may potentially lead to disseminated intravascular coagulation (DIC) and subsequent organ damage. Endothelial dysfunction can be notably impacted by exosomal signals from various cells, thereby contributing to ongoing inflammation and coagulation. Conversely, exosomes from endothelial progenitor cells (EPCs) play a crucial role in facilitating vascular repair and reducing endothelial permeability, effectively countering the vascular effects of sepsis. This underscores the significant role of exosomes in the development of coagulopathy during sepsis. PS phosphatidylserine, TF tissue factor, HMGB1 high mobility group box 1, ARA arachidonic acid, IL-10 interleukin 10, MMP9 matrix metallopeptidase 9

In the initial phase of host-pathogen interaction, the activation of coagulation plays a key role in linking coagulation and innate immunity, a process recently termed “immunothrombosis” [77]. Sepsis is particularly associated with intense activation of the coagulation system, which may lead to disseminated intravascular coagulation (DIC), clinically manifested by microvascular thrombosis and hemorrhage, the latter being caused by the excessive consumption of clotting factors and platelets. Approximately 35% of sepsis patients experience DIC, and the current treatment primarily focuses on symptomatic support [77]. Despite numerous attempts, other treatment approaches have yet to show statistical success in large-scale clinical trials [78]. Exosome measurement could potentially aid in the early quantification of DIC risk, which is very useful for patients in the early stage of sepsis [79]. Figure 2 illustrates the various pathways that exosomes contribute to the development of sepsis-induced coagulopathy.

The exposition of phosphatidylserine (PS) on the exosomal surface exhibits direct procoagulant activity. PS, a cellular membrane phospholipid, facilitates the functionality of tissue factor (TF) and thrombin, acting as a primary initiator for the coagulation cascade. In sepsis, procoagulant exosomes predominantly originate from monocytes, endothelial cells, and platelets [80–82]. Given that TF is the main trigger of the coagulation cascade and PS acts as a catalyst for coagulation component activation, this exosome subset is a plausible vector for propagating the procoagulant phenotype during sepsis. Hematogenous-derived TF-positive exosomes also implicate the potentially overarching prothrombotic environment of DIC [83]. Elucidating this mechanism could unveil novel therapeutic targets while intervening in the genesis or functionality of these exosomes might delineate new trajectories for managing sepsis-accompanied DIC.

Noteworthy, PMN-derived exosomes play a pivotal role in thrombogenesis. These vesicles facilitate the transfer of arachidonic acid to platelets, which then utilize cyclooxygenase-1 to synthesize thromboxane A2, a potent vasoconstrictor and platelet aggregator. This biochemical cascade is crucial for inducing PMN extravasation and promoting thrombus formation, highlighting a novel aspect of PMN-platelet crosstalk in thrombosis [84]. In sepsis, the administration of PMN-derived exosomes to murine models has been shown to increase the bacterial load, diminish PMN recruitment, upregulate IL-10 expression, and ultimately, higher mortality rates [85]. These suggest that PMN-derived exosomes may have a detrimental effect on the host defense against sepsis, potentially by altering the immune response and disrupting the delicate balance required for effective pathogen clearance. Moreover, PMN-derived exosomes may induce endothelial cell senescence through redox-sensitive pathways, contributing to a pro-inflammatory and pro-coagulant environment that exacerbates the severity of the host response [86]. Furthermore, these effects are further complicated by exosomal miR-146a mediated interplay between oxidized low-density lipoprotein-treated macrophages and PMNs, downregulating superoxide dismutase 2 expression in PMNs. This downregulation leads to an overproduction of ROS and the formation of NETs, which significantly increases the risk of thrombosis [87].

### Platelet-derived exosomes link coagulation and inflammation

Overactivation of platelets is implicated in organ damage during sepsis through various mechanisms, including enhanced recruitment of immune cells and inflammation, promotion of microvascular bed thrombus formation, and direct cytotoxic effects mediated by platelet-derived microparticles. In vitro studies have shown that exosomes generated from activated platelets contain abundant activated factor IX, factor Va, and binding sites for factor VIII, as well as augment the activity of factor Xa and thrombin [88, 89]. Patients at increased risk of thromboembolic complications (e.g., those undergoing cardiac surgery) have elevated levels of circulating platelet-derived exosomes, which are positively correlated with myocardial infarction incidence [90]. In wound blood collected directly from the pericardial cavity of patients undergoing cardiac surgery, levels of platelets and erythrocyte-derived exosomes are elevated [91]. When added to normal plasma, these exosomes can promote thrombin generation through interaction with TF and factor VII, thus exhibiting procoagulant properties [92].

In sepsis, platelet-derived exosomes may promote vascular cell apoptosis and increase the risk of thrombus formation through NADPH oxidase activity [93]. Platelet-derived exosomes induce caspase-3 activation and apoptosis in endothelial cells by generating superoxide, NO, and peroxynitrite, resulting in endothelial barrier dysfunction [94]. During septic shock, platelet-derived exosomes promote the formation of NETs through HMGB1, miRNAs (miR-15b-5p and miR-378a-3p), as well as Akt/mTOR autophagy pathway, exacerbating DIC and organ dysfunction [74]. Exosomes could serve as useful biomarkers for DIC in sepsis patients. Early detection of specific exosomes, such as CD31^+^ or CD105^+^ exosomes, may help predict the occurrence of DIC, thus providing earlier and more effective management and treatment options for sepsis patients [79, 95].

In conclusion, exosomes significantly contribute to the development of sepsis-induced coagulopathy through various pathways. Their interaction with different cellular components and the subsequent modulation of inflammatory and coagulation responses underscores their pivotal role in the complex pathophysiology of sepsis. The multifaceted nature of exosomes, which are derived from various cell types including PMNs, endothelial cells, and platelets, plays a crucial role in either propagating or mitigating the coagulation abnormalities observed in sepsis.

### Endothelial cell-derived exosomes and endothelial dysfunction

During sepsis, endothelial cells amplify the immune response and activate the coagulation system. They are both a target and source of inflammation, bridging local and systemic immune responses [96]. Confronted with local infections, leukocytes and platelets adhere to the endothelial surface, migrating to sites with significant bacterial proliferation. In this defensive environment, exosomes secreted by endothelial cells are capable of engaging in adhesive interactions with circulating leukocytes, setting the stage for inflammation and potentially immunological assaults against vascular injury [97, 98]. Dysfunctional exosomes from endothelial cells enhance the formation of inflammatory macrophages through NF-кB and IL-1β signalings [99].

The systemic and endothelial activation orchestrated by LPS and thrombin/CD40 ligand (CD40L) escalates the proportion of exosomes laden with CD40L and matrix metalloproteinase (MMP)10 both intravascularly and extravascularly. A surge in circulating CD40L and MMP10, particularly in septic patients with elevated thrombin levels, correlates with a heightened mortality rate [82]. Mast cell-derived exosomes augment the secretion of plasminogen activator inhibitor-1 from endothelial cells, thereby modulating the coagulation and fibrinolytic mechanisms [100]. Additionally, endothelial cell-derived exosomes play an important role in preserving blood fluidity and modulating vascular bioactivity by catalyzing the conversion of plasminogen to plasmin [101]. However, exosomes derived from endothelial progenitor cells (EPCs) hold a therapeutic promise, potentially ameliorating microvascular dysfunction, curtailing vascular leakage, enhancing organ functionality, and bettering sepsis outcomes through the delivery of miR-126 and miR-21-5p [102, 103]. Moreover, the transference of miR-199a-3p by EPC-derived exosomes may prevent endothelial cell apoptosis as well as glutathione depletion, ROS production, lipid peroxidation, and iron accumulation, thus opening a promising avenue for the alleviation of sepsis-induced endothelial dysfunction [104].

## Exosome-mediated intercellular crosstalk in organ dysfunction

In sepsis-related organ dysfunction, exosome-mediated intercellular crosstalk plays a critical role. Initially, specific immune cells release exosomes loaded with pro-inflammatory signals, thereby exacerbating inflammation and leading to further organ damage [105, 106]. As the condition progresses, a different set of immune cells begins to produce exosomes with anti-inflammatory and reparative signals, aiding in the healing process [107]. Moreover, injured tissue cells contribute to this crosstalk by releasing their own exosomes, which help modulate the immune response and facilitate the shift from inflammation to repair [66]. This dynamic exchange of exosomes among varied cell types underscores their pivotal role in the complex interplay of inflammation and healing in sepsis-induced organ dysfunction.

### In ALI

**Fig. 3:**
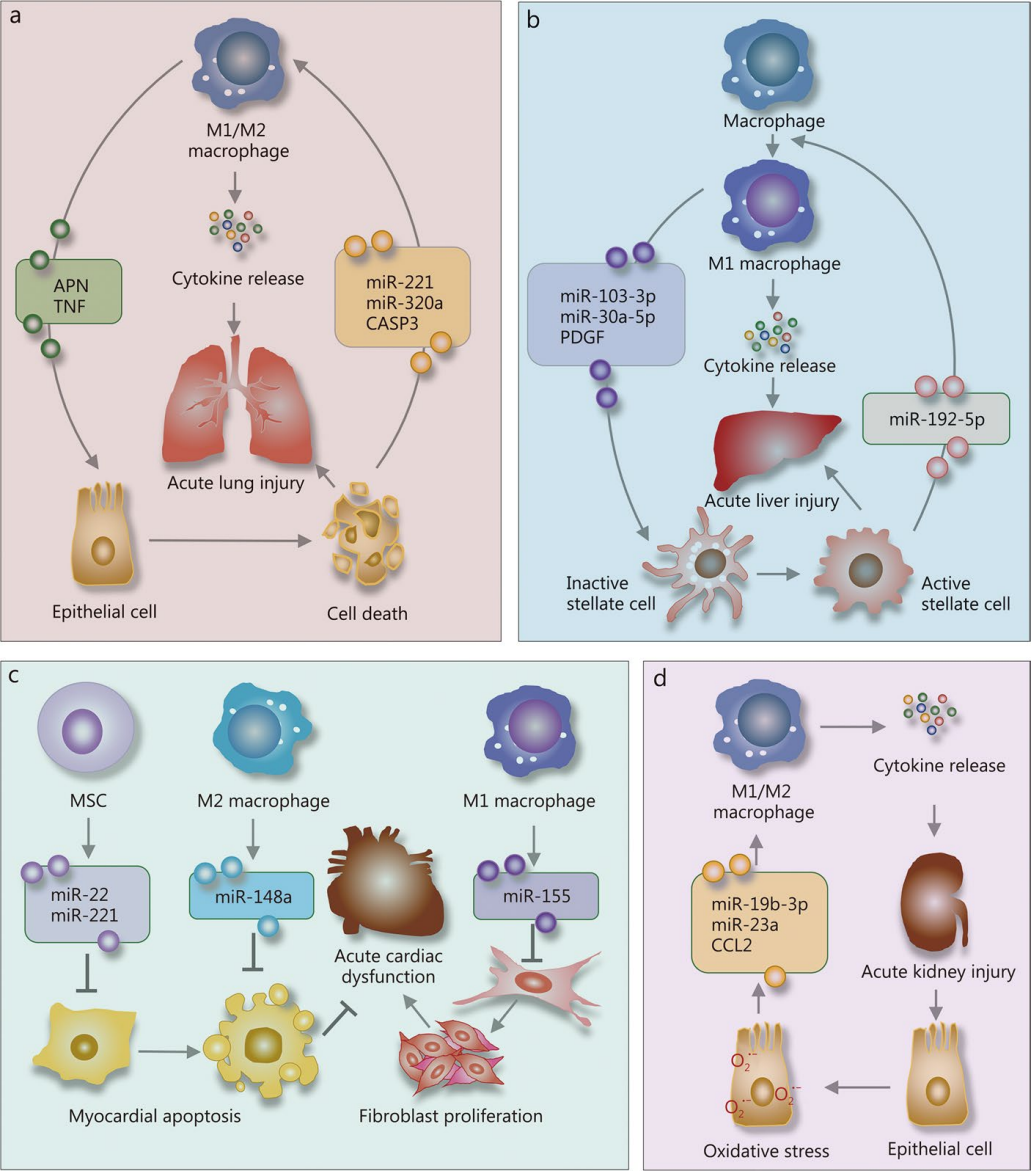
Exosome-mediated intercellular crosstalk in organ dysfunction. Exosomes mediate crucial intercellular communication between releasing and recipient cells, playing a significant role in the progression of sepsis-related organ dysfunction. **a** In acute lung injury, epithelial cells play a crucial role by releasing exosomes abundant in proteins and miRNAs, which induce macrophages to polarize towards the M1 phenotype, amplifying pulmonary inflammation. On the other hand, M1 macrophage-derived exosomal APN/CD13 and TNF exacerbate lung injury by promoting programmed cell death in lung epithelial cells. **b** In acute liver injury, hepatocytes release exosomes containing miR-192-5p, prompting macrophages to adopt the M1 phenotype. This polarization leads to the release of inflammatory mediators like iNOS, IL-6, and TNF-α into the hepatic microenvironment, worsening hepatic cell dysfunction. Additionally, exosomes from M1 macrophages containing miR-103-3p promote the proliferation and activation of hepatic stellate cells, further contributing to liver injury. **c** In acute cardiac dysfunction, exosomes from M1 macrophages containing miR-155 inhibit fibroblast proliferation, thus contributing to cardiac damage. Conversely, exosomes from M2 macrophages containing miR-148a suppress inflammasomes, reducing myocardial injury. MSCs further protect the heart by releasing exosomes laden with miR-22 and miR-221, which counteract cardiomyocyte apoptosis. **d** In acute kidney injury, tubular epithelial cells damaged by oxidative stress secrete exosomes with miR-19b-3p and miR-23a, inducing macrophages to polarize into the M1 phenotype. This exacerbates renal inflammation and contributes to the progression of kidney damage. APN/CD13 aminopeptidase N, TNF tumor necrosis factor, miRNAs microRNAs, M1 type 1 macrophage, M2 type 2 macrophage, iNOS inducible nitric oxide synthase, IL-6 interleukin 6, TNF-α tumor necrosis factor-α, MSCs mesenchymal stem cells, CASP3 caspase-3, PDGF platelet-derived growth factor, CCL2 C-C motif chemokine ligand 2

During episodes of ALI, exosomes released within the lung tissue exert a significant influence on the inflammatory milieu. These exosomes play an important role in modulating the inflammatory response both locally and systemically by promoting the activation and recruitment of immune cells, as well as orchestrating the release of both pro-inflammatory and anti-inflammatory cytokines [108]. It is worth noting that these exosomes may be derived from diverse pulmonary cell types, including vascular endothelial and alveolar epithelial cells [109]. Their functionality is likely contingent upon the informational molecules they carry and the state of the recipient cells (Fig. 3a).

Research has indicated a significant increase in the number of exosomes in bronchoalveolar lavage fluid following exposure to sterile and infectious stimuli [110, 111]. In the case of sterile stimuli, alveolar type I epithelial cells are the predominant source of exosomes, whereas, under infectious conditions, exosomes are primarily derived from alveolar macrophages (AMs) [111]. In both non-infectious and infectious models of ALI, exosomes have been shown to enhance the recruitment of macrophages and differentially regulate the production of cytokines by AMs, the release of inflammatory mediators, and the expression of TLRs [112, 113].

Conversely, some studies have identified a lung-protective role for exosomes released by EPCs through the modulation of immune cell phenotypes. Endotoxin-induced release of exosomes from EPCs have been shown to carry lncRNAs that promote macrophage M2 polarization, thereby ameliorating organ damage caused by sepsis [114]. Exosomes from AdMSCs have been reported to mitigate ALI in septic mice by suppressing macrophage release of inflammatory factors [115]. Moreover, IL-25 from pulmonary epithelial cells has been observed to inhibit the release of exosomes by macrophages, thus alleviating lung injury [116]. It is evident that the impact of exosomes on macrophages is dependent on the molecules they contain, the origin of the parent cells, as well as their pathological and physiological states.

Pro-inflammatory exosomes released by immune cells exacerbate ALI in sepsis. Macrophage-derived exosomal aminopeptidase N (APN/CD13) contributes to the aggravation of sepsis-induced ALI by regulating necroptosis of lung epithelial cells [117]. AMs play a crucial role in the clearance of inhaled bacteria [118]. Following bacterial infection, AMs are activated into a pro-inflammatory phenotype, which enables them to phagocytose bacteria more effectively and release inflammatory cytokines, chemokines, and ROS [119]. Additionally, exosomes released by AMs facilitate communication between activated and resting AMs, further propagating the inflammatory cascade [120].

In addition, PMN-derived exosomes are rich in PMN elastase and can perform active proteolysis unimpeded by antiproteases, leading to damage and cell apoptosis of extracellular matrix (ECM), further exacerbating lung tissue damage [121]. Furthermore, PMN-derived exosomes, which are abundant in MMP9, contribute to the cleavage of desmoglein-2 — a pivotal protein for cell-cell adhesion — and subsequent degradation at cell junctions. This process undermines cellular cohesion and disrupts the integrity of the tissue structure, resulting in extensive tissue damage and impairment of epithelial barrier function [122].

In the complex milieu of pulmonary injury, exosomes serve as pivotal mediators of intercellular crosstalk between immune and tissue cells, thereby influencing the trajectory of organ tissue damage, either contributing to its exacerbation or facilitating its amelioration [108, 117]. This dichotomy presents a compelling avenue for therapeutic intervention, modulating the phenotype of pro-inflammatory exosomes may attenuate the inflammatory cascade and tissue damage. Furthermore, the reduction of macrophage uptake of exosomes could also hold therapeutic potential. Such investigations are crucial to determine how the modulation of exosome phenotype or the regulation of their uptake by macrophages could potentially be employed in the treatment of lung injury.

### In acute liver injury

In sepsis, exosomes secreted by differentiated macrophages contribute to liver injury. A previous study showed that exosomes derived from LPS-induced macrophages act on hepatocytes, activating the NOD-like receptor family, pyrin domain containing 3 (NLRP3) inflammasome pathway, subsequently leading to liver injury [123]. Exosomes from LPS-treated THP-1 macrophages carrying miR-103-3p promote the proliferation of hepatic stellate cells (HSCs) and induce liver fibrosis [124]. Bone marrow-derived Ly6C^hi^ macrophages dominate the macrophage population in the injured liver. This subtype of liver macrophages is pro-inflammatory and crucial for the activation of HSCs by producing pro-fibrotic factors such as transforming growth factor-β (TGF-β) and platelet-derived growth factor [125]. Ly6C^hi^ macrophages undergo a phenotypic switch to a Ly6C^lo^ subset during the resolution of fibrosis, characterized by upregulation of MMPs and downregulation of inflammatory cytokines [126]. Activated HSCs are the primary effector cells of liver fibrosis, inducing liver injury by producing ECM. Relaxin-activated macrophages release exosomes containing miR-30a-5p, which target and inhibit apoptosis signal-regulating kinase 1, thereby promoting the upregulation of peroxisome proliferator-activated receptor-gamma expression, enhancing the sensitivity of activated HSCs to relaxin-mediated inactivation, and thus counteracting fibrosis and promoting the restoration of liver function [127].

Hepatocytes can also release exosomes that activate or polarize macrophages in the liver. For instance, after lipotoxic injury, hepatocyte-secreted exosomes containing miR-192-5p can induce polarization of macrophages towards the M1 phenotype, releasing inducible nitric oxide synthase, IL-6, and TNF-α into the liver microenvironment and exacerbating hepatocyte dysfunction [128]. These hepatocyte-secreted exosomes also contain TNF-related apoptosis-inducing ligands, which can promote the inflammatory phenotype of receptor macrophages [129].

The complexity of liver injury induced by sepsis, mediated through exosomes, underscores the intricate interplay between immune regulation and cellular communication (Fig. 3b). Ongoing research in this field is imperative for developing targeted interventions that could significantly reduce the morbidity and mortality associated with sepsis and its hepatic complications.

### In acute cardiac dysfunction

During cardiac inflammation, circulating monocytes are recruited to the heart tissue where they differentiate into macrophages. These macrophages secrete exosomes that contribute to cardiac injury by impairing vascular remodeling and exacerbating myocardial damage [130]. The macrophage-derived exosomes containing miR-155 inhibit fibroblast proliferation and promote fibroblast inflammation during cardiac injury, leading to impaired cardiac repair after myocardial infarction [131]. Exosomes from M2 macrophages, carrying miR-148a, alleviate myocardial ischemia/reperfusion injury through downregulation of thioredoxin-interacting protein (TXNIP) and inhibition of the TLR4/NF-κB/NLRP3 inflammasome signaling pathway [107].

A recent study has shown that MSCs can reduce ischemic myocardial cell injury through their exosomes containing miR-22, which targets methyl CpG binding protein 2, thereby reducing cell apoptosis [132]. Additionally, MSC-derived exosomes with miR-221 exert cardioprotective effects against myocardial apoptosis by inhibiting the expression of p53 upregulated modulator of apoptosis [133]. The exosomes derived from MSCs can increase ATP levels, reduce oxidative stress, and activate the PI3K/Akt pathway, thereby enhancing myocardial vitality and preventing adverse remodeling after myocardial ischemia/reperfusion injury [134]. In case of septic shock, platelet-derived exosomes induce endothelial cell apoptosis and circulate in patients, consequently promoting myocardial dysfunction [135].

These findings highlight the multifaceted roles of exosomes in the heart, wherein they can either exacerbate damage or offer protective mechanisms, depending on their source and the molecules they carry (Fig. 3c). Utilizing modulation of exosomal content or signaling pathways presents an opportunity to alleviate cardiac complications associated with sepsis. The identification of exosomal molecules that activate protective pathways in cardiac cells may lead to the development of targeted therapies aimed at reducing myocardial damage. However, realizing this potential necessitates a detailed understanding of the mechanisms by which exosomes mediate intercellular crosstalk at the molecular level. Further clinical trials are also essential to validate these findings and translate them into effective therapeutic strategies.

### In acute kidney injury (AKI)

TECs are the most abundant cell type in the kidney and play a crucial role in pathological renal injuries. Tubulointerstitial inflammation is a hallmark of various acute and chronic kidney diseases, and emerging evidence suggests that TEC-derived exosomes communicate with renal macrophages, leading to kidney damage. For instance, exosomes from TECs carrying miR-19b-3p have been shown to induce polarization of M1 macrophage, triggering renal inflammation (Fig. 3d) [136]. Additionally, TEC-derived exosomes containing C-C motif chemokine ligand 2 can activate macrophages and induce AKI [137].

Similarly, exosomes released from TECs under hypoxic conditions, enriched with miR-23a, have been found to induce M1 macrophage polarization and tubulointerstitial inflammation by promoting local inflammatory responses [71]. Taken together, the inflammation associated with AKI is largely regulated by communication between TECs and renal resident macrophages. TEC-derived exosomes induce polarization of M1 macrophage and inflammation, thereby initiating or exacerbating tissue damage.

### In central nervous system (CNS) dysfunction

In sepsis, CNS is considered both a critical trigger point and a victim of the condition. The brain, being a target in septic shock, is involved in the propagation of immune-inflammatory dysregulation and alterations in cerebral hemodynamics [138]. The potential mechanisms underlying sepsis-induced cerebral dysfunction include increased permeability of the blood-brain barrier and heightened activity of MMPs, loss of tight junction proteins, and degeneration of endothelial cells [139]. This process facilitates the influx of inflammatory and toxic mediators, such as exosomes carrying inflammatory factors, into the brain ultimately leading to cerebrovascular damage [12].

Research indicates that circulating exosomes act as neuroinflammatory mediators in systemic inflammation. Transfusion of serum-derived exosomes from LPS-challenged mice into other mice enhances the activation of microglial and astrocytic, and increases the expression of inflammatory cytokines in the brain [140]. In sepsis, choroid plexus epithelial cells sense peripheral inflammation and transmit signals to the CNS by releasing exosomes enriched with miR-146a and miR-155 into the cerebrospinal fluid, thereby transferring the pro-inflammatory message to recipient brain cells [141]. By mediating inflammatory responses and crossing the blood-brain barrier, exosomes serve as key facilitators of the neuro-inflammatory processes that contribute to cerebral dysfunction in sepsis. Furthermore, their impact extends beyond the CNS, potentially influencing systemic immune responses and resulting in the broader immunological dysregulation observed in sepsis.

In summary, the intricate mechanisms of exosome-mediated intercellular crosstalk underscore the complexities of immune regulation and cellular communication in the context of sepsis-induced organ dysfunction. This crosstalk is pivotal in orchestrating the pathological state across various organs, including but not limited to the lung, heart, liver, and kidney. Exosomes serve as vectors for both pro-inflammatory and anti-inflammatory signals to modulate processes such as tissue repair, fibrosis, and immune cell activation. The ongoing research in this field is critical for the development of targeted therapeutic interventions aimed at reducing the incidence and mortality associated with sepsis and its sequelae in organ function. Strategies that focus on modulating exosome release or modifying their cargo composition hold great promise for attenuating acute tissue damage and enhancing recovery. These may include the use of exosome inhibitors, the creation of exosomes loaded with therapeutic agents, or altering how cells absorb exosomes. Such approaches present novel opportunities for sepsis treatment and management of its associated complications.

## Therapeutic potentials of exosomes in sepsis

### The potential value of exosomes as biomarkers

The clinical application potential of exosomes as diagnostic and prognostic biomarkers has garnered increasing attention due to several key factors. (1) Exosomes exhibit high specificity as they contain a diverse array of proteins, RNA, and other molecules that directly originate from their parent cells. This enables exosomes to accurately reflect the physiological and pathological states of these cells, making them promising candidates for early disease diagnosis, prognosis assessment, and monitoring treatment outcomes. (2) Exosomes possess the ability for early detection of disease, allowing timely intervention. (3) Their non-invasive or minimally invasive sampling method significantly reduces patient discomfort and complication risks compared to conventional tissue biopsies. (4) Exosomes exhibit stability and detectability in bodily fluids, showing resistant to enzymatic degradation. This makes them convenient for storage, transport, and utilization in clinical diagnosis settings. (5) By providing comprehensive biological information through their diverse biomolecules, exosomes contribute to a deeper understanding of disease mechanisms and guide personalized treatment. (6) Their potential for dynamic monitoring and therapeutic guidance through real-time tracking capabilities that inform physicians about disease progression and treatment efficacy, leading to more accurate treatment plans for patients. Consequently, human-derived exosomes isolated from bodily fluids (such as whole blood, plasma, urine, and ascites) and tissue biopsies are emerging as promising biomarkers for clinical diagnostics [142].

Beyond propelling exosomes towards mainstream clinical application, high-throughput methods for isolating exosomes from liquid and solid biopsies may further unveil the systemic and localized functions of these extracellular vesicles. The prognostic field of exosomes is a focal point in over 100 completed and ongoing clinical trials, witnessing a rapid expansion [8]. Similarly, circulating exosomes have shown immense potential as diagnostic biomarkers for sepsis. A recent clinical study encompassing 220 patients revealed a significant correlation between high levels of plasma exosomes and increased severity of organ failure as well as mortality in patients with critical sepsis [143]. Furthermore, studies have noted that the biological effects of exosomes on recipient cells largely depend on their cargo including miRNAs or the proteins they carry [144, 145]. With advancements in proteomics technologies, they are now widely applied in the detection of circulating exosomal biomarkers, new possibilities are unveiled for understanding the variations and functions of exosomes in different disease states [19, 117].

Significant progress has been achieved in the field of sepsis by using the proteomic profiles of plasma exosomes. Through the acquisition and analysis of proteomic mass spectrometry and targeted proteomics, scientists have successfully identified exosomal proteins that are closely linked to the progression of sepsis, thus providing new biomarkers for its diagnosis and prognosis [117]. In another study, the assessment of circulating de novo DNA methyltransferase (DNMT) mRNA within exosomes in the plasma of patients admitted to the ICU has emerged as an innovative diagnostic method for septic shock. The findings suggest that the levels of circulating DNMT mRNA, with the total count of exosomes, can effectively diagnose septic shock and may also hold prognostic significance [146].

Exosomal nucleic acids are also being explored as biomarkers for sepsis. A study has shown that compared to healthy volunteers, patients with sepsis have elevated levels of specific miRNAs (miR-276-3p, miR-21-5p, and miR-193a-5p) in their plasma exosomes, which correlate with the severity of the disease [147]. Similarly, circulating exosomal miR-193a-5p and miR-542-3p can distinguish patients with community-acquired pneumonia or sepsis from healthy volunteers, and the expression level of exosomal miR-1246 is positively correlated with the severity of sepsis [148].

In summary, the exploration of exosomes as biomarkers in sepsis opens a promising avenue in medical research. These findings not only enhance our understanding of the molecular mechanisms underlying sepsis but also lay a foundation for developing novel diagnostic and therapeutic strategies. Investigating the proteomic and miRNA content of exosomes holds the potential to identify clinically relevant biomarkers, which could offer more accurate guidance in managing and treating septic patients. However, current clinical research on this topic is relatively limited. Future studies should focus on larger-scale, multicenter clinical trials to validate and further explore exosomal biomarkers. Such research is essential to advance personalized medicine and provides feasible options for precision treatment in clinical settings.

### The therapeutic potential of exosomes

The therapeutic potential of exosomes is rapidly expanding in the fields of regenerative medicine and drug delivery. MSCs actively promote regeneration in damaged tissues through enhancing in situ cellular regeneration and immunomodulation, fostering angiogenesis, and inhibition of cell death by cell-to-cell contact effects as well as via exosomes [34]. Recently, over 900 clinical trials globally have employed MSCs for the treatment of various diseases, including bone/cartilage repair, diabetes, cardiovascular diseases, immune-related disorders, and neurological diseases [149]. However, the majority of intravenously injected MSCs are sequestered in filtering organs, with insignificant homing to injury sites. Exosomes purified from MSCs overcome some limitations associated with MSC-based therapies, such as allogeneic immune rejection and premature cell differentiation. These results suggest that exosomes hold superior potential for the clinical treatment of sepsis and organ injury. Furthermore, MSC-derived exosomes can be engineered to carry specific proteins or genes that promote cellular functions and tissue repair, thus making them ideal candidates for regenerative medicine treatments.

Sepsis, in particular, may benefit from the application of exosome therapy. Exosomes can act as vehicles to deliver anti-inflammatory agents and repair molecules directly to the sites of tissue damage. This targeted approach may alleviate the detrimental inflammatory cascades and promote healing processes. Exosomes derived from MSC-enhance angiogenesis due to their specific protein and transcript composition associated with vasculogenic and proliferative functions. It has been further elucidated that the protein content within MSC-derived exosomes mediates angiogenesis by modulating the NF-κB signaling pathway [150]. Moreover, MSC-derived exosome miRNAs, such as miR-21, miR-23a, miR-125b, and miR-145, contribute to the suppression of myofibroblast formation by inhibiting TGF-β2/SMAD2 signaling, thereby reducing scar formation during the wound healing process [151]. Currently, several strategies aimed at enhancing the release of exosomes from MSCs are under exploration. Hypoxia has been shown to promote exosome secretion from MSCs, thereby improving cardiac tissue repair in a mouse model of myocardial infarction [152].

The ability of MSC-derived exosomes to fuse with and deliver their cargo directly into recipient cells renders them a promising tool for modulating pathological processes at the cellular level. In AKI, specific miRNAs carried by MSC-derived exosomes, such as miR-15a, miR-15b, and miR-16, attenuate the accumulation of pro-inflammatory macrophages in the kidneys by inhibiting the expression of CX3C chemokine ligand 1 [153]. Concurrently, MSC-derived exosomes carrying miRNAs suppress TLR signaling, thereby preventing macrophages from being activated by mitochondria they have phagocytosed [154]. Additionally, MSC-derived exosomes can increase ATP production by augmenting enzymes required for glycolysis, reduce oxidative stress, improve cellular metabolism, and exert cardioprotective effects [134]. This modulation of immune response underscores a crucial mechanism by which MSC-derived exosomes exert their protective roles in acute organ injury. Therefore, it is expected that MSC-derived exosomes will alleviate acute organ injury.

In conclusion, MSC-derived exosomes are at the forefront of regenerative medicine, with the potential to significantly transform the treatment approaches for a wide array of organ pathologies present in critically ill patients, including but not limited to sepsis, AKI, myocardial infarction, traumatic brain injury, and liver failure, etc. Their remarkable adaptability, low immunogenicity, and inherent targeting properties establish them as a highly promising platform for the development of innovative therapeutic interventions. The ongoing advancement in research and clinical trials is progressively uncovering the full capabilities of these exosomes, setting a solid foundation for their integration into critical care practices.

### Advantages of exosomes in therapeutic delivery systems

Compared to cell therapies, a principal advantage of exosomes is their suitability for specialized and large-scale production. Several platforms are currently under development for scaled-up immunoprecipitation production and purification of exosomes [155]. Recently described methodologies even allow for the rapid, automated collection and surface modification of exosomes on microfluidic devices [156]. For internal cargo modification, several methods have been established, including sonication, electroporation, and passive loading [157].

Multiple advantages have propelled the clinical transition from cells to exosomes. Exosomes possess a phospholipid bilayer, ensuring convenient storage and exhibiting enhanced stability through freeze-thaw cycles, as well as preventing rapid degradation in vivo [158]. They remain unaffected by the inflammatory microenvironment and evade immune polarization. Due to their biocompatibility, stability, targeting capability, and scalability, exosomes are ideal candidates for drug development and delivery. Rich in adhesion molecules and signaling entities, exosomes can target specific cells and stimulate uptake [159]. The presence of transmembrane CD47 allows exosomes to avoid immune rejection through the CD47-signal regulatory protein alpha (SIRPα) “don’t eat me” signal [160]. This immune evasion mechanism contributes to an extended circulation time for exosomes compared to free drugs or cell therapies that are more susceptible to immune clearance. Beyond their prolonged half-life, exosomes also exhibit stronger cellular targeting and absorption capabilities than free drug delivery [161]. Their nanoscale structure facilitates passage through biological barriers to reach distant injury sites. Moreover, their modifiable cargo makes them ideal vehicles for molecular delivery, with a lower likelihood of immune rejection, rendering them attractive therapeutic agents.

Utilizing exosomes as therapeutic agents and delivery vehicles offers several potential advantages over cells and traditional drug delivery systems such as liposomes and nanoparticles. (1) The smaller size of exosomes may minimize entrapment in small capillaries following systemic infusion, improving the targeted delivery of therapeutics to diseased sites. (2) Exosomes present a complex mixture of factors targeting different therapeutic pathways and act synergistically to enhance therapeutic functions, as opposed to using single factors. (3) The natural cellular origin of exosomes enhances the ability to genetically modify the origin cells to produce exosomes with overexpressed agents, resulting in improved efficacy, biocompatibility, and reduced immunogenicity [5, 25].

B cell-derived exosomes have been shown to act as vehicles for the delivery of exogenous miRNA-155 inhibitors to macrophages, effectively reducing the secretion of the pro-inflammatory cytokine TNF-α [162]. Furthermore, the inherent epitope presentation on B cell-derived exosomes provides a more efficient and stable approach to activating the immune system. This enhances T-cell responses and antibody production, bolstering the body’s defense mechanisms against pathogens [53]. These insights pave new avenues for vaccine development and immunotherapy, utilizing exosomes as a platform for therapeutic intervention.

The potential of exosomes as drug delivery vehicles is a promising area of research and development in the field of drug delivery, with the potential to offer new, more effective ways of delivering therapeutic agents to specific cells or tissues, minimizing systemic exposure and associated side effects. Although challenges remain in the large-scale production, isolation, and characterization of exosomes to ensure consistency and efficacy in their therapeutic use, advances in bioprocessing and analytical technologies are helping to address these obstacles, laying the groundwork for the broader adoption of exosome-based therapies in the clinic.

## Conclusions

In this review, we extensively explored the critical role of exosomes in sepsis and inflammatory organ injury, particularly their involvement in intercellular communication and organ dysfunction. Our discussion highlights how exosomes modulate immune responses, metabolic reprogramming, coagulopathy, and endothelial integrity, thereby contributing to the complexity of sepsis pathophysiology. This understanding bridges the gap between the biological functions of exosomes and their potential therapeutic applications.

While the current focus on the therapeutic potential of exosomes is promising, this conclusion aims to reconnect these applications with the fundamental rationale of the review. Exosomes, as intricate mediators in sepsis, offer a dual role in both exacerbating and alleviating the disease’s progression. Their involvement in various cellular processes makes them not only potential biomarkers for early diagnosis and prognosis of sepsis but also opens avenues for novel therapeutic strategies. The exploration of exosomes as carriers for targeted drug delivery, for example, is grounded in their natural ability to facilitate intercellular communication, which is crucial in the pathogenesis of sepsis and other multiple organ system failure scenarios.

As research in this field continues to evolve, the potential of exosomes in the realm of sepsis treatment becomes increasingly evident. This review emphasizes the necessity for further investigation into the multifaceted roles of exosomes, to harness their capabilities for more effective, targeted, and safe therapeutic interventions in sepsis and other inflammatory organ failure-related disorders.

## Data Availability

Not applicable.

## Abbreviations

AdMSC-Exos: Adipose-derived mesenchymal stem cell-derived exosomes
AKI: Acute kidney injury
Akt-mTOR: Protein kinase B-mammalian target of rapamycin
ALI: Acute lung injury
AMs: Alveolar macrophages
APCs: Antigen-presenting cells
APN/CD13: Aminopeptidase N
cGAS/STING: Cyclic GMP-AMP synthase/stimulator of interferon genes
CNS: Central nervous system
DCs: Dendritic cells
DIC: Disseminated intravascular coagulation
DNMT: DNA methyltransferase
ECM: Extracellular matrix
EPCs: Endothelial progenitor cells
HIF-1α: Hypoxia-inducible factor-1α
HMGB1: High mobility group box 1
HSCs: Hepatic stellate cells
ICU: Intensive care unit
IFN: Interferon
IL: Interleukin
ILVs: Intraluminal vesicles
IRF3: Interferon regulatory factor 3
LPS: Lipopolysaccharide
MHC: Major histocompatibility complex
miRNAs: microRNAs
MMP: Matrix metalloproteinase
MSC-Exos: Mesenchymal stem cell-derived exosomes
MSCs: Mesenchymal stem cells
MVBs: Multivesicular bodies
NADPH: Nicotinamide adenine dinucleotide phosphate
ncRNAs: Non-coding RNAs
NETs: Neutrophil extracellular traps
NF-κB: Nuclear factor-kappa B
NLRP3: NOD-like receptor family, pyrin domain containing 3
Nrf2/HO-1: Nuclear factor erythroid 2-related factor 2/heme oxygenase-1
OXPHOS: Oxidative phosphorylation
PI3K/Akt: Phosphoinositide 3-kinase/protein kinase B
PMN: Neutrophils
PS: Phosphatidylserine
RAG: Recombination-activating gene
ROS: Reactive oxygen species
S1P/SK1/S1PR1: Sphingosine-1-phosphate/sphingosine kinase 1/sphingosine-1-phosphate receptor 1
SHIP1: SRC homology 2-containing inositol phosphatase 1
SIRPα: Signal regulatory protein α
SOCS1: Suppressor of cytokine signaling 1
STATs: Signal transducer and activator of transcription proteins
TECs: Tubular epithelial cells
TF: Tissue factor
TGF-β: Transforming growth factor-β
Th1: Type 1 helper T cells
Th2: Type 2 helper T cells
TLR: Toll-like receptor
TNF-α: Tumor necrosis factor-α
TRAF1: Tumor necrosis factor receptor-associated factor 1
Treg: Regulatory T
V(D)J: Variable (V), diversity (D), and joining (J)
